# A Treatise on the Diseases of Infancy and Childhood

**Published:** 1896-07

**Authors:** 


A TREATISE ON THE DISEASES OF INFANCY AND CHILDHOOD. By J. LEWIS SMITH, M. D., Clinical Professor of Diseases of Children in the Bellevue Hospital Medical College, New York. New, eighth edition, thoroughly revised and rewritten and much enlarged. Octavo of 983 pages, with 273 illustrations and four full-page plates. Cloth, $4.50; leather, $5.50. New York and Philadelphia: Lea Brothers & Co., Publishers. 1896.

A medical text-book whose success warrants the issuance of an eighth edition hardly demands a review. It may be considered a classic. The new edition of this work has been enlarged by nearly one hundred pages. The text shows careful revision, some chapters having been entirely rewritten, as for instance that on rickets. The chapter on diphtheria marks one great advance in medicine in the last five years, the tentative views of 1890 contrasting forcibly with the positive statements of 1895.

Several new chapters appear, among them one on myxedema, a second on blood diseases, a third on diseases and injuries of bone and a fourth on diseases of the heart. These and other additions give the book a completeness as a work on pediatrics which the older editions certainly lacked, and brings up to date a treatise which has long been regarded standard. M. J. F.

